# GasanalyzeR: advancing reproducible research using a new R package for photosynthesis data workflows

**DOI:** 10.1093/aobpla/plae035

**Published:** 2024-06-20

**Authors:** Danny Tholen

**Affiliations:** Department of Integrative Biology and Biodiversity Research, Institute of Botany, the University of Natural Resources and Life Sciences, Vienna, 1180 Vienna, Austria

**Keywords:** Chlorophyll fluorescence, gas exchange, R package, reproducible research, stable carbon isotopes

## Abstract

The analysis of photosynthetic traits has become an integral part of plant (eco-)physiology. Many of these characteristics are not directly measured, but calculated from combinations of several, more direct, measurements. The calculations of such derived variables are based on underlying physical models and may use additional constants or assumed values. Commercially available gas-exchange instruments typically report such derived variables, but the available implementations use different definitions and assumptions. Moreover, no software is currently available to allow a fully scripted and reproducible workflow that includes importing data, pre-processing and recalculating derived quantities. The R package gasanalyzer aims to address these issues by providing methods to import data from different instruments, by translating photosynthetic variables to a standardized nomenclature, and by optionally recalculating derived quantities using standardized equations. In addition, the package facilitates performing sensitivity analyses on variables or assumptions used in the calculations to allow researchers to better assess the robustness of the results. The use of the package and how to perform sensitivity analyses are demonstrated using three different examples.

## Teaser

GasanalyzeR, a new software tool, helps analyse data related to photosynthesis to achieve consistent and reproducible results. Many physiologically relevant variables related to photosynthesis require calculations based on models and assumptions. GasanalyzeR offers standardized equations to ensure consistent results, even with data from different instruments. It also lets you perform sensitivity analyses, revealing how the conclusions depend on measurements and assumptions. The tool streamlines analysis workflow by importing and pre-processing data for effortless integration into R. This allows you to leverage the power of R for advanced analysis and insightful visualizations.

## Background

The assessment of photosynthetic characteristics has emerged as a crucial component of plant physiological research. Commercially available infrared gas analysers, often combined with chlorophyll fluorometers and isotope analysers, allow researchers to delve into specific aspects of photosynthetic physiology in great detail (Long and Hällgren 1985; Field *et al.* 1989; Long *et al.* 1996; Long and Bernacchi 2003; Welles and McDermitt 2005; Baker 2008; Ubierna *et al.* 2018). These instruments typically track the concentrations of carbon dioxide (CO_2_) and water vapour (H_2_O) of air flowing through a leaf chamber, and also monitor gas flow rates, temperatures, air pressure and light levels. However, many physiologically relevant characteristics cannot be directly measured by such instruments, but are derived using mathematical descriptions of the biophysical and biochemical processes involved, which inherently include assumptions or simplifications (Farquhar *et al.* 1980; von Caemmerer and Farquhar 1981; Long and Bernacchi 2003; Baker 2008; Ubierna *et al.* 2018). These calculated or derived quantities provide information about biochemical properties of leaves (Long and Bernacchi 2003), are used in growth analyses (Wilson 1981; Poorter 2002), can help assess acclimation and stress resistance (Farquhar *et al.* 1989; Evans and Poorter 2001; Ainsworth and Long 2005; Chaves *et al.* 2011; Yamori *et al.* 2014), and inform crop, ecosystem or even global carbon cycle models (Sitch *et al.* 2008; Rogers 2014; Rogers *et al.* 2017; Wu *et al.* 2019).

Examples of such derived, physiologically relevant variables are the rate of assimilation (*A*), the rate of evapotranspiration (*E*), stomatal conductance to H_2_O (*g*_sw_) and the CO_2_ mole fraction in the substomatal cavities (*C*_i_). Under steady-state conditions, the former two variables are derived from more direct measurements (the gas flow rates and mole fractions of CO_2_ and H_2_O) in combination with a constant (the leaf area enclosed in the chamber). The latter two variables (*g*_sw_ and *C*_i_) are calculated from the already mentioned measured and derived variables, together with several additional constants related to the boundary layer conductance of the leaf in the chamber and the ratio of stomatal conductance between the upper and lower leaf sides (LI-COR Biosciences Inc. 2022*a*). The calculation also relies on a model for the ternary diffusion of CO_2_ and H_2_O through the stomata and boundary layer, and on an empirical relation to estimate the water vapour mole fraction inside the leaf (von Caemmerer and Farquhar 1981). These models use additional assumptions. For example, the contribution of gas fluxes through the leaf cuticle is typically ignored, and the water vapour pressure in the substomatal cavity is assumed to be equal to the saturation vapour pressure at leaf temperature. Measurements suggest that this is not always the case (Boyer and Kawamitsu 2011; Tominaga *et al.* 2018; Márquez 2021; Márquez *et al.* 2021; Wong *et al.* 2022).

A basic set of equations to calculate derived variables has been incorporated in the firmware of commercial gas-exchange instruments, but such implementations do not allow users to modify many of the assumptions discussed above. Moreover, additional variables are typically calculated when gas exchange is combined with chlorophyll fluorescence (Bellasio *et al.* 2016), or with measurements of stable carbon isotope compositions (Ubierna *et al.* 2018). An example of a derived variable resulting from such combined protocols is the mesophyll conductance (*g*_m_), describing the ease of CO_2_ diffusion between the substomatal airspace and the site of carboxylation in the chloroplasts. The calculation of *g*_m_ also depends on additional constants and model assumptions.

Gas-exchange instruments store measurements as tabular data that are easily imported into spreadsheet applications. Data files stored by the LI-6400XT and LI-6800 instruments (LI-COR Biosciences Inc 2011, 2022*a*) even include equations that allow recalculation of many derived quantities. This is convenient in case the user wants to modify some constants after the measurement (such as the leaf area, or the ratio of stomatal conductance between the adaxial and abaxial side of the leaf). Protocols for gas exchange have assumed or recommend that the data are imported in spreadsheet applications (Evans *et al.* 2014; Douthe *et al.* 2018), and subsequent analysis of gas-exchange data also sometimes relies on such software (Sharkey *et al.* 2007; Bellasio *et al.* 2016).

The interactive approach (such as copy-and-paste or drag-and-drop operations) taken by spreadsheet applications does not document how a certain result was obtained and, therefore, it has been argued that such workflow forms a significant barrier to fully reproducible research (Peng 2011). Such interactive operations and the tendency to write formulas as cell references make spreadsheets hard to audit (Powell *et al.* 2008). Scripted approaches using documented code result in a traceable protocol for every step performed between raw initial data and the final results of the analysis (Peng 2011; Stinziano *et al.* 2021). As a result, such approaches allow for a uniform and reproducible treatment of data and are especially convenient when processing large datasets in the context of high-throughput experiments. Although more time and skill may be required to initially develop a scripted workflow, the resulting pipeline can be used for the analysis of similar data in the future.

**Figure 1.**
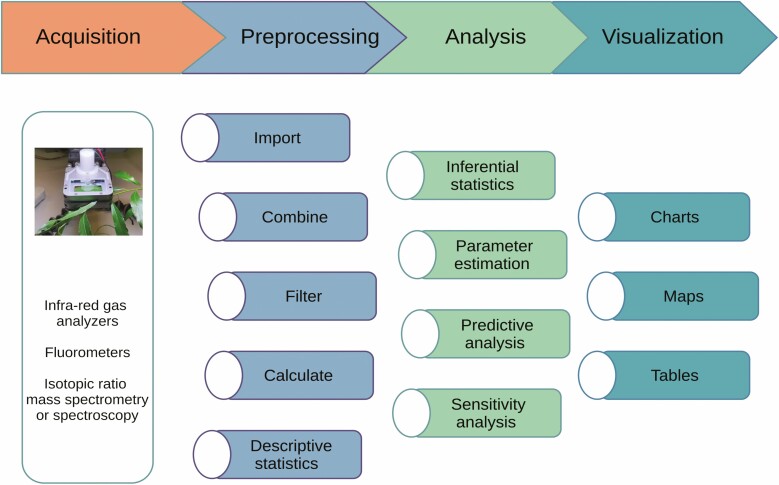
Typical analysis workflow for quantification of photosynthetic traits. In the first step, measurements of one or more specific aspects of the photosynthetic process are acquired by scientific instruments. During the pre-processing stage, data from multiple replicates or from different instruments are combined, outliers or irrelevant measurements are filtered, and calculations for additional derived quantities may take place. The pre-processed data are then further analysed using statistical methods or in combination with physiological models. Finally, charts, maps and tables are produced to illustrate the results. The R language is increasingly being used for the analysis and visualization stages, and gasanalyzer will facilitate its use especially during the pre-processing stage.

The R programming language (R Core Team 2023) is one of the tools that can be used to implement scripted workflows and is gaining traction in eco-physiologal research (Stinziano *et al.* 2021; Liu *et al.* 2022). While the implementation presented here utilizes R, the underlying functionalities are designed to be language-agnostic. Modern data science workflows and integrated development environments facilitate seamless integration with other programming languages (Giorgi *et al.* 2022). For photosynthesis research, the use of R is currently limited to parameter estimation and simulating data (Duursma 2015; Muir 2019; Gregory *et al.* 2021; Stinziano *et al.* 2021). To the best of my knowledge, no comprehensive tool implementing a scripted approach for pre-processing gas exchange and fluorescence data is currently available (Fig. 1).

A standardized nomenclature for reporting gas-exchange results was recently proposed (Ely *et al.* 2021), which is an important step towards making results more reproducible and comparable. Since different instruments often use different names or abbreviations, a translation table was included in the proposal, but is only of limited use since the instruments sometimes use different definitions for similar variables. For example, the Walz GFS-3000 instrument stores a variable with the difference between reference and sample mole fractions without applying a zero-offset (match) correction, whereas instruments manufactured by LI-COR store a similar variable that already includes this correction (Heinz Walz GmbH 2019; LI-COR Biosciences Inc. 2022*a*). Additional differences in derived variables reported by the instruments result from taking into account over-pressure in the leaf chamber (only for the LI-6800; LI-COR Biosciences Inc. 2022*a*), from taking into account the effect of boundary layer conductance and the ratio of stomatal conductance between the upper and lower sides of the leaf (both effects are assumed negligible in the calculations implemented in the firmware of the Walz GFS-3000; Heinz Walz GmbH 2019), and from the type of equation used for calculating the saturation water pressure (GFS-3000 uses a Goff-Gratch equation, whereas the other instruments use a modified Buck equation; Goff and Gratch 1946; Buck 1981). The model equations currently implemented in all instruments assume that the humidity in substomatal cavities equals the saturation vapour pressure and that the conductance through the cuticle is negligible. Both assumptions have been questioned and alternative descriptions were proposed in recent literature (Márquez *et al.* 2021; Wong *et al.* 2022).

Although LI-COR instruments can store data in a spreadsheet to recalculate a limited number of derived variables from the measurements (LI-COR Biosciences Inc 2022*a*), an instrument-independent reference implementation would be a valuable asset for reproducible research. The R package gasanalyzer aims to provide such an implementation for variables related to gas exchange, chlorophyll fluorescence and stable carbon isotope discrimination. In addition, the package aids researchers in importing and pre-processing photosynthesis data. Importantly, methods and documentation are provided that allow users to rapidly assess the impact of biases in assumed or measured variables, or of different calculation methods. Although the package cannot cover all possible derived quantities that may be of interest, it is designed to be extensible and available under a free and open-source license.

## Implementation

The software is implemented as a collection of data, documentation and source code for use with the R programming language for statistics and graphics (R Core Team 2023). It is made available under the GNU General Public License allowing users to freely use, modify and share the code as long as the changes are shared under the same license. The latest development version of gasanalyzer can be found on GitLab (https://gitlab.com/plantphys/gasanalyzer), and a stable version is regularly published through the Comprehensive R Archive Network (CRAN, https://cran.r-project.org/package=gasanalyzer). Dependencies on packages other than those included in the base R install were kept to a minimum to avoid potential dependency problems and maintainability issues. Nevertheless, a number of external packages are currently required: to ensure the validity of equations, units are enforced in all calculations by the R package units (Pebesma *et al.* 2016). The tidyxl (Garmonsway 2022), jsonify (Cooley 2022) and xml2 (Wickham *et al.* 2023) packages are used to import gas-exchange and calibration data, and the stringi package (Gagolewski 2022) was used for faster and more portable internal string processing. In addition, gasanalyzer relies on tibble (Müller and Wickham 2023) for neater presentation of the data, and vctrs (Wickham *et al.* 2023) for speeding up several slow steps in the data processing. Installing gasanalyzer from CRAN (by typing install.packages('gasanalyzer') on the R command line) will also automatically install all required dependencies.

After importing and pre-processing by gasanalyzer, data can subsequently be analysed, visualized and further processed using the wide range of tools available for the R environment. Table 1 lists all methods implemented in the package together with a short description. Detailed information on how to use these methods is provided in the software documentation. Resilient coding principles (Stinziano *et al.* 2021) were implemented by ensuring a consistent nomenclature and style, and programming a modular and extendable application interface. Currently, importing files from four different types of gas analysers is supported (LI-COR LI-6400/LI-6400XT, LI-COR LI-6800, Walz GFS-3000 and PP Systems CIRAS-4). To facilitate further processing and analysis, all imported data are converted into a ‘tidy’ format (every variable forms a column and every observation forms a row; Wickham 2014). Variable names used in the files outputted by the different instruments are converted to a standardized nomenclature [for a complete list, **see****Supporting Information—Table S1**] on import.

**Table 1.** List and brief description of the different methods provided by the gasanalyzer package. More detailed usage information is available in the software documentation.

Method name	Description
create_equations	Create a list of equations for use with the calculate() method. See also Table 2.
export_ess_dive	Export to the ESS-DIVE reporting format for leaf-level gas-exchange data.
get_factory_cals	Return a matrix with factory calibration information for given instrument serial numbers and calibration dates.
import_factory_cals	Import instrument-specific factory calibration files from a folder.
modify_equations	Modify an existing list of equations with user-specified equations.
permutate	Expand a data frame with all possible combinations of the given values in a specific column. Used for setting up sensitivity analyses.
read_6400_txt	Read 6400 or 6400XT text files and create an R tibble with gas-exchange data.
read_6800_equations	Read gas-exchange equations directly from 6800 xlsx files and output them as a list.
read_6800_txt	Read 6800 text files and create an R tibble with gas-exchange data.
read_6800_xlsx	Read 6800 xlsx files and create an R tibble with gas-exchange data.
read_gfs	Read GFS-3000 data files and creates an R tibble with gas-exchange data.
read_ciras4	Read CIRAS-4 csv files and creates an R tibble with gas-exchange data.
read_gasexchange	Read gas-exchange data from a text file exported by gasanalyzer.
recalculate	Recalculate gas-exchange data based on a given list of equations.
var2label	Render gasanalyzer variables or values using mathematical notation **[****see Supporting Information—Table S1****]**.
write_gasexchange	Write a gas-exchange tibble to a tab-separated text file.

Ideally, a standardized nomenclature should be used throughout the research field (Stinziano *et al.* 2021) and was recently proposed for reporting results related to gas exchange (Ely *et al.* 2021). However, modern analysers report a much larger number of variables (>150) than those included in the existing proposal. In addition, similar variables may be reported by different components of an instrument (e.g. light intensity and spectral information can be reported by multiple connected light sources or sensors, multiple sensors can report leaf or chamber temperatures) and variables may be calculated using different approaches (such as fluxes calculated for steady-state or non-steady-state conditions). This requires a much more detailed naming scheme. Some variables are instrument specific but still need to be standardized because they are used in calculations of derived quantities. Rather than opting for a mixture of naming schemes, a more detailed nomenclature was adopted **[see****Supporting Information—Table S1****]**, originally based on the categories and variable names currently used by the instrument that reports the largest number of variables (LI-COR Biosciences Inc. 2022*a*). However, variables were made more specific if ambiguous (e.g. Offset was changed to TleafOffset to make more clear which offset it refers to), and variable names were made consistent and usable in programming languages (i.e. the first letter of each compound word in a variable is always capitalized, unless the word refers to, or is part of a mathematical symbol. Greek letters are always spelled out). Only letters, digits, periods and underscores part of the ASCII character set are allowed in variable names, as these can be used for valid language objects in most programming languages, and they can be easily used on most keyboards. A method was provided (export_ess_dive(), see Table 1) to export data using the proposed standard nomenclature for reporting and interaction with other software (Ely *et al.* 2021). In addition, the package supports rendering commonly used variables in mathematical notations (var2label()), which can conveniently be used for plotting the results [for the symbol list, see **Supporting Information—Table S1**].

Gas-exchange instruments report a large number of variables that are not directly measured but calculated by combining several of the measured quantities. Often, additional information that is not directly measured by the instrument is necessary for these calculations. Instrument-specific constants (such as those related to the spectral quality of the used light source, or the leaf chamber properties that determine the boundary layer conductance) are taken from the data files if available, or set automatically if the type of instrument is known. The calculation of derived quantities typically also requires biological or protocol-specific variables. If such variables are related to gas exchange, and likely to be constant over several observations, they are found in the Const category **[see****Supporting Information—Table S1****]**. These values are not determined by the instrument and may be related to a specific protocol used (e.g. the amount of leaf area enclosed in the leaf chamber), or be related to a biological characteristic that has been either assumed or determined by additional experiments (e.g. the ratio of stomatal conductances between the upper and lower side of the leaf, or the cuticular conductance to water vapour).

If equations for calculating derived quantities were stored with the data (in the case of xlsx files), these can be automatically imported by gasanalyzer. The equations can subsequently be used to recalculate the data if required. For instruments where equations are not stored with the data, it is still possible to recompute the data using equations defined in the R package (see Table 2). A function is provided that constructs a list of equations based on user-supplied keywords (see Table 2 for an overview and description of the equation sets that are currently supported). The default set of equations recalculates variables using the widely used theory described by von Caemmerer and Farquhar (1981). Based on instrument documentation (LI-COR Biosciences Inc. 2022*a*; Heinz Walz GmbH 2019; PP Systems 2024), this set can be complemented by adding an instrument keyword that will add vendor-specific equations (typically required for calculating the boundary layer conductance surrounding a leaf in a specific gas-exchange chamber, for leakage corrections, or for calculating variables influenced by the used light spectrum). The R package allows users to modify assumptions in gas-exchange theory. For example, the equations used to calculate the saturation water vapour pressure can be modified (Buck1981, Buck1996 or GoffGratch1946), the model described by Márquez *et al.* (2021) can be used by adding the cuticular_conductance keyword, and the relative humidity the substomatal cavity (Wong *et al.* 2022) can be modified by providing a value for Const.RHi. The equation list can also be manually modified or extended by the user. The software automatically resolves possible dependencies between different equations, so they are evaluated in the correct order. Because gas-exchange measurements are often complemented by measurements of chlorophyll fluorescence or carbon isotope ratios, equations to derive *g*_m_ from such measurements have also been implemented (using the gm_fluorescence and d13C keywords). For the LI-6800 instrument, the package supports importing calibration files, and the raw and O2_correction keywords allow recalculation of the gas concentrations from low-level instrument data (LI-COR Biosciences Inc 2022*a*).

**Table 2.** Sets of equations for derived variables defined in gasanalyzer, with references to literature or instrument manuals describing these calculations. Depending on the data or purpose of the analysis, one or more of these sets can be combined to recompute variables. In addition, custom equations can be added to the analysis. **See****Supporting Information—Table S1** for a detailed explanation of all variables.

Name of equation set	Description
default	Default set with all derived quantities in the gas-exchange (GasEx) and chlorophyll fluorescence (FLR) categories **[see****Supporting Information—Table S1****]**. These equations have been described by von Caemmerer and Farquhar (1981) and LI-COR Biosciences Inc (2022*a*).
ciras4	Light absorptance (*α*, LeafQ.alpha) and the conversion between photon flux density and irradiance (LeafQ.Conv) is calculated by taking into account the effect of the different light sources that can be used with the CIRAS-4 instrument (PP Systems 2024).
gfs3000	Boundary layer conductance (*g*_bw_, GasEx.gbw) and light sensor (*Q*_in_, LeafQ.Qin) calculations adjusted for the default chamber of the GFS-3000 instrument (Heinz Walz GmbH 2019).
gfs3000_light_bot	Requires gfs3000, but modifies *Q*_in_ (LeafQ.Qin) to indicate that the bottom light sensor of the default chamber was used to quantify the light intensity incident on the leaf (Heinz Walz GmbH 2019).
li6400	Definitions for boundary layer conductance, temperature and light-related variables (*g*_bw_, GasEx.gbw; *R*_abs_, GasEx.Rabs; *T*_air,Cnd_, GasEx.TairCnd; *α*, LeafQ.alpha) specific to the LI-6400 / LI-6400XT instruments (LI-COR Biosciences Inc 2011).
li6800	Definitions for boundary layer conductance, fan, leakage and light related variables (*g*_bw_, GasEx.gbw; *R*_abs_, GasEx.Rabs; *T*_air,Cnd_, GasEx.TairCnd; *α*, LeafQ.alpha; *Q*_in_, LeafQ.Qin; LeafQ.Conv; Leak.Fan; Leak.CorrFact; *C*_a_, GasEx.Ca) specific to the LI-6800 instrument (LI-COR Biosciences Inc 2022*a*).
cuticular_conductance	Replaces the equations related to CO_2_ and H_2_O conductance and substomatal CO_2_ (*g*_tw_, GasEx.gtw; *g*_sw_, GasEx.gsw; *g*_tc_, GasEx.gtc; *C*_i_, GasEx.Ci) with versions that take into account cuticular conductance (Márquez Antivilo 2021; Márquez *et al.* 2021, 2023). Requires manually specifying the cuticular conductance to water (*g*_cw_, Const.gcw) and CO_2_ (*g*_cc_, Const.gcc).
GoffGratch1946	Replaces or adds equations related to the calculation of the saturated water pressure of the leaf (GasEx.SVPleaf) and chamber air (GasEx.SVPcham). Only takes temperature into account. Based on Goff and Gratch (1946).
Buck1981	Replaces or adds equations related to the calculation of the saturated water pressure of the leaf (GasEx.SVPleaf) and chamber air (GasEx.SVPcham). Takes temperature and pressure into account, and is based on the Buck equation (Buck 1981). The default calculations (also used by LI-COR and PP Systems) are derived from this equation but do not account for air pressure (LI-COR Biosciences Inc. 2022*a*; PP Systems 2024).
Buck1996	Replaces or adds equations in the default set related to the calculation of the saturated water pressure of the leaf (GasEx.SVPleaf) and chamber air (GasEx.SVPcham). Takes temperature and pressure into account. Based on the modified Buck equation (Buck 1996). This version provides the greatest accuracy.
match	Takes a previously stored offset between sample and reference analysers into account when recalculating water and CO_2_ mole fractions ([CO_2_]_s_, Meas.CO2s; [H_2_O]_s_, Meas.H2Os) as described by LI-COR Biosciences Inc. (2022*a*), Heinz Walz GmbH (2019) and PP Systems (2024). The corrected mole fractions are already stored in the data and therefore this calculation is usually not required. However, these equations are needed when recalculating mole fractions from lower level data (see the raw and O2_correction sets).
raw	Currently only implemented for the LI-6800 because low-level instrument data are required. Recalculates CO_2_ and H_2_O mole fractions from such low-level variables ([CO_2_]_r_, Meas.CO2r; [H_2_O]_r_, Meas.H2Or; [CO_2_]_a_, Meas.CO2a; [H_2_O]_a_, Meas.H2Oa) as described by LI-COR Biosciences Inc. (2022*a*). Requires storing raw data and the availability of factory calibration files. Requires and enables the match set.
O2_correction	Currently only implemented for the LI-6800 (LI-COR Biosciences Inc 2022*a*) and GFS-3000 (K. Siebke, Heinz Walz GmbH, pers. comm.). Recalculates CO_2_ and H_2_O mole fractions ([CO_2_]_r_, Meas.CO2r; [H_2_O]_r_, Meas.H2Or; [CO_2_]_a_, Meas.CO2a; [H_2_O]_a_, Meas.H2Oa) at a potentially different oxygen concentration ([O_2_], Const.Oxygen). For the LI-6800, this requires loading of factory calibration files (import_factory_cals()). Requires and automatically enables the match set.
gm_fluorescence	Adds derived variables for mesophyll conductance (*g*_m,flr_, FLR.gm) and chloroplast CO_2_ mole fractions (*C*_c,flr_, FLR.Cc) based on gas exchange and chlorophyll fluorescence variables (Harley *et al.* 1992). It is strongly recommended to first calibrate the electron transport rates (*J*_F_) in the FLR.ETR column (van der Putten *et al.* 2018). In addition, requires manually adding columns for the respiration rate in the light (*R*_L_, Const.RL), and the CO_2_ photo-compensation point (Γ^*^, Const.GammaStar).
d13C	Adds derived variables for *ξ* (d13C.xi), *a*ʹ (d13C.ap), *e*ʹ (d13C.ep), *t* (d13C.t), Δ_i_ (d13C.Deltai), Δ_o_ (d13C.Deltao), Δ_i_–Δ_o_ (d13C.DeltaiDeltao), *A*/*pC*_a_ (d13C.A_pCa), *g*_m,13C_ (d13C.gm), and *C*_c,13C_ (d13C.Cc) based on the stable carbon isotope discrimination model for C_3_ plants (Farquhar and Cernusak 2012; Evans and von Caemmerer 2013). Requires additional columns with data on the carbon isotope composition in sample and reference air (δ^13^C-CO_2,s_, d13CMeas.delta13CO2s; δ^13^C-CO_2,r_, d13CMeas.delta13CO2r), in the air where the plants were grown (δ^13^C-CO_2,g_, d13CConst.delta13CO2g), and values for *R*_L_ (Const.RL) and Γ^*^ (Const.GammaStar).
d13C_dis	Requires the d13C set, but modifies the modelled carbon isotope discrimination, Δ_i_ (d13C.Deltai), and *g*_m,13C_ (d13C.gm) using the assumption that the carbon pools of respiration and assimilation are disconnected (as described by Busch *et al.* 2020).
d13C_e_Busch2020	Requires the d13C set, but modifies the calculation of the effective respiratory fractionation, *e*ʹ (d13C.ep), to better take into account the effect of the growth conditions (Busch *et al.* 2020). Additionally requires a value for the observed discrimination against ^13^CO_2_ under growth conditions (d13CConst.Deltag).

Recomputing gas-exchange data is useful when a change in an assumed or independently measured variable is made, but can also be very helpful when performing sensitivity analyses. For the latter, a function is provided (permutate(), see Table 1) that varies a specific assumed or measured variable over a specified range, while keeping all other data constant. Making a comparison between different models (i.e. sets of equations such as those given in Table 2) is also supported.

### Data used in the examples

Poplar trees (*Populus canadensis* Moench cv. ‘Robusta’) were grown between May and August from cuttings in a greenhouse on campus. Twelve-litre pots filled with garden soil were used, watered daily and fertilized weekly using liquid 8-8-6 NPK fertilizer. Fully expanded leaves of 4-month-old plants were used for gas-exchange experiments. For the first example, assimilation rate was measured at different CO_2_ levels using an LI-6800 gas analyser (LI-COR Biosciences Inc., Lincoln, NE, USA) equipped with a fluorometer (6800-01/A). The instrument was connected to tank air with a certified gas mixture (1 % O_2_ in 99 % N_2_). For the second example, the relation between assimilation rate and CO_2_ levels was measured for leaves of three different plants using a Walz GFS-3000 portable gas analyser (Walz GmbH, Effeltrich, Germany). The ratio of stomatal conductance was measured using an LI-600 Porometer/Fluorometer (LI-COR Biosciences Inc.) and the ratio of stomatal densities was determined by counting the number of stomata within a field of view at 20× magnification using a light microscope (Leica DM5500 B, Leica Microsystems GmbH, Wetzlar, Germany). For the final example, data for a tobacco leaf (*Nicotiana tabacum*, cv. Wisconsin 38) were used. The gas-exchange data were acquired by an LI-6400 and ^13^CO_2_/^12^CO_2_ ratios were obtained using a Los Gatos Research CCIA-36d isotope analyser. Details of the growth conditions and experimental design are given in Tholen *et al.* (2012).

All data and code used to generate the figures in this article are available at the GitLab repository for the gasanalyzer package (https://gitlab.com/plantphys/gasanalyzer).

## Results and Discussion

The R package gasanalyzer provides methods for importing data from different instruments and presents the data in a consistent format with a standardized nomenclature [for a complete list, **see****Supporting Information—Table S1**]. The package can be used not only to pre-process data for analysis but also to recompute quantities commonly used in photosynthesis. Equations for these computations can be imported from vendor-supplied files (if available), but alternative sets of equations described in the literature are also included in the software package (e.g. equations based on the gas-exchange theory by von Caemmerer and Farquhar 1981; or those based on Márquez *et al.* 2021). Note that the software does not introduce new theoretical approaches to calculate derived quantities, but currently only implements equations that have already been published or are available from instrument vendors on request (see the references given in Table 2).

To demonstrate the abilities of the R package, three detailed examples are provided below. The first example demonstrates how to import, filter and recompute LI-6800 data. It also shows how to visualize the results. The second example shows how to import multiple GFS-3000 files and perform a sensitivity analysis to analyse the robustness of the results. The final example shows how to import data from an LI-6400 that has been combined with isotopic measurements. It demonstrates how different sets of model equations affect the results, and it showcases how the package integrates with existing functionality in R for analysing data. A detailed step-by-step guide to analyse real-world data is provided in a vignette that is part of the documentation of the package and can be viewed by running vignette('gasanalyzer', package = 'gasanalyzer') from within an R environment.

### Importing data from the LI-6800 and recomputing derived variables

**Figure 2.**
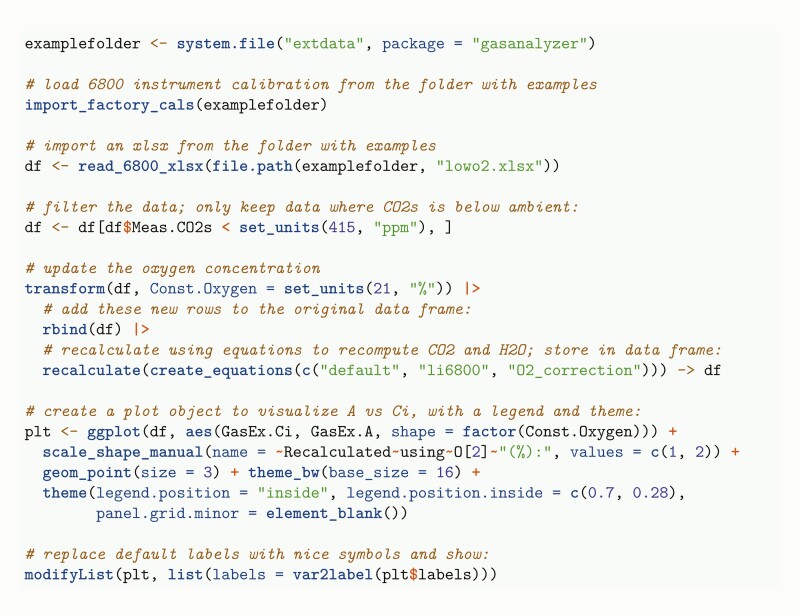
Example of data import, filtering, recalculating, and visualizing a plot.

Fig. 2 shows the program code required to import an LI-6800 xlsx file, filter the data, recompute derived quantities and visualize the results. Recomputing of derived quantities is sometimes necessary when correcting mistakes, or in case more accurate information for some of the constants or variables becomes available after the measurements. In the example, the oxygen concentration stored in the data was updated, and derived quantities were recomputed (this specific recalculation is currently not implemented in the vendor-provided xlsx file).

The calibration of infrared gasanalyzers is affected by the oxygen concentration because the width of the absorption bands used to derive the gas mole fractions depends on all gasses present in the mixture (Bunce 2002; Loriaux and Welles 2004; Welles and McDermitt 2005). The instruments are typically calibrated using ambient oxygen concentrations so that no corrections are needed when measuring under such conditions. For measurements done at oxygen concentrations other than 21 %, several vendors support measuring or manually entering the used oxygen concentration in the instrument firmware, and this value is subsequently used to adjust the reported mole fractions (LI-COR Biosciences Inc. 2011, 2022*a*; Heinz Walz GmbH 2019). The calibration factors used for this adjustment depend on the instrument type, and default values stored in the instruments may not always be accurate (Tholen *et al.* 2012). For the LI-6400/LI-6400XT, a software program is available that can be used to correct previously collected data when new calibration factors are determined, or when an erroneous oxygen value was entered during the measurements (LI-COR Biosciences Inc. 2023). For the Walz GFS-3000 instrument, a spreadsheet for this purpose is available from the instrument vendor. The functionality has been integrated in gasanalyzer and allows oxygen concentrations to be taken into account when recomputing gas mole fractions. This feature makes it also possible to investigate the effect of this correction for typical gas-exchange experiments. Entering the correct oxygen concentration for experiments done under non-photorespiratory conditions has been recommended because it affects several derived quantities (Pons *et al.* 2009).

**Figure 3.**
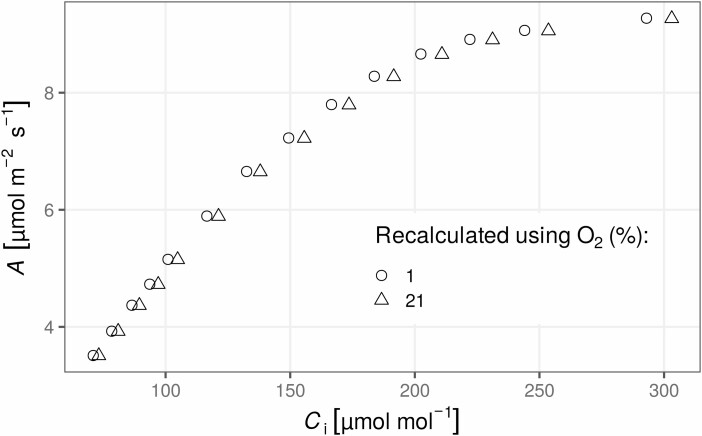
The relationship between net assimilation (*A*) and the substomatal mole fraction of CO_2_ in air (*C*_i_) calculated at two different atmospheric oxygen levels. The plot was created using the code presented in Fig. 2. The original data were measured at 1 % O_2_, and failing to take this into account in the calculations would have resulted in an overestimation of *C*_i_.

The results shown in Fig. 3 indicate that not accounting for the oxygen concentration affects the estimates of *C*_i_, but does not matter much for *A*. The calculation of *C*_i_ depends not only on the measurement of CO_2_ mole fractions but also on the measurement of water vapour. Thus, although the effect of the oxygen concentration on CO_2_ mole fractions is typically small (Loriaux and Welles 2004; Welles and McDermitt 2005), the influence on water vapour mole fraction (and therefore on derived variables such as *g*_sw_ and *C*_i_) is much bigger (Bunce 2002).

The package documentation extends this example and shows how to calibrate estimates of the electron transport rate obtained by chlorophyll fluorescence measurements to those obtained directly from gas-exchange measurements at low oxygen concentrations (Pons *et al.* 2009; van der Putten *et al.* 2018).

### Sensitivity analysis with gas-exchange data

The previous example highlighted the usefulness of being able to test the potential effect of an error in measurements or assumptions on the experimental results. Accurate determination of photosynthetic characteristics requires careful calibration and configuration of the used instruments, but in many cases, systematic errors cannot be completely avoided. Thus, interpretation of experimental results requires awareness of the relative importance of bias in different variables. In addition, differences in results may also originate from different assumptions made in models underlying the calculation of gas-exchange variables. For example, Lamour *et al.* (2022) used a new theory developed by Márquez *et al.* (2021) combined with a sensitivity analysis to show that conductance of water vapour through the leaf cuticle affected parameter estimates for the biochemical model for C_3_ photosynthesis (Farquhar *et al.* 1980). There is little information available in the literature on the relative effect of systematic errors in different measured or assumed variables on derived quantities related to photosynthesis. Therefore, functionality was added to the gasanalyzer package to perform sensitivity analyses on variables or equations.

This second example shows the result of a sensitivity analysis using gas-exchange data measured on three different poplar leaves using a GFS-3000 instrument. The analysis explored four potential sources of bias on three physiologically relevant output variables (*A*, *g*_sw_ and *C*_i_). The first source of bias is related to a potential difference in the stomatal conductance between the upper and lower sides of the leaf. The ratio between the conductances (*K*) can be determined using additional measurements (LI-COR Biosciences Inc. 2022*b*) and can be entered in the instrument firmware before the measurements take place (LI-COR Biosciences Inc. 2011, 2022*b*; PP Systems 2024). However, very few studies have mentioned whether this ratio was taken into account (Toro *et al.* 2019), and it remains unclear how much, if at all, this setting affects the results. It is worthwhile to mention that the ratio of conductances is not the same as the ratio of the stomatal density (Anderson and Briske 1990; Pearson *et al.* 1995; Márquez *et al.* 2023). For the poplar leaves used for this analysis, the ratio of stomatal densities was 0.7, but the ratio of stomatal conductances (*K*) was typically around 0.5 (which was used as the default value for the analysis). The second source of bias is related to accounting for cuticular conductance (*g*_cw_) as described by Márquez *et al.* (2021). The effect of this conductance is currently not taken into account by commercially available gas-exchange instruments. Because measurements of cuticular conductance remain challenging (Márquez *et al.* 2021), analysing its potential effect on experimental results is useful. Although typical values for *g*_cw_ are likely below 10 mmol m^−2^ s^−1^, its effect on gas exchange becomes more pronounced when stomata close (Márquez *et al.* 2021; Lamour *et al.* 2022). To make sure the analysis covered a realistic range (*g*_cw_ between 0 % and 20 % of the total conductance), the default value for the analysis shown here was put at the relatively high value of 25 mmol m^−2^ s^−1^ (10 % of the average total leaf conductance). The effect of the relative humidity in the stomatal cavities (RH_i_) was the third source of error analysed. This factor is also difficult to measure experimentally and is likely to vary with the vapour pressure deficit at leaf temperature (VPD_leaf_). The average VPD_leaf_ for the analysed leaves was 0.73 kPa, suggesting RH_i_ is above 95 % (Wong *et al.* 2022), which was the default value used for the analysis (allowing a range between 90 % and 100 % RH_i_ to be analysed). The final source of bias considered is related to the measurement of leaf temperatures. Leaf chamber design and the use of thermocouples may result in biased measurements for leaf and air temperature (Mott and Peak 2011; Still *et al.* 2019; Garen *et al.* 2022). Therefore, the effect of a systematic error in leaf temperature on derived quantities was included in the analysis. These four sources of possible bias are compared with a potential systemic error in the H_2_O calibration of the infrared gas analyser. Such a deviation in the measurement of the gas mole fractions is unlikely and easily avoided by regular calibration, but it is included here as a comparison.

**Figure 4.**
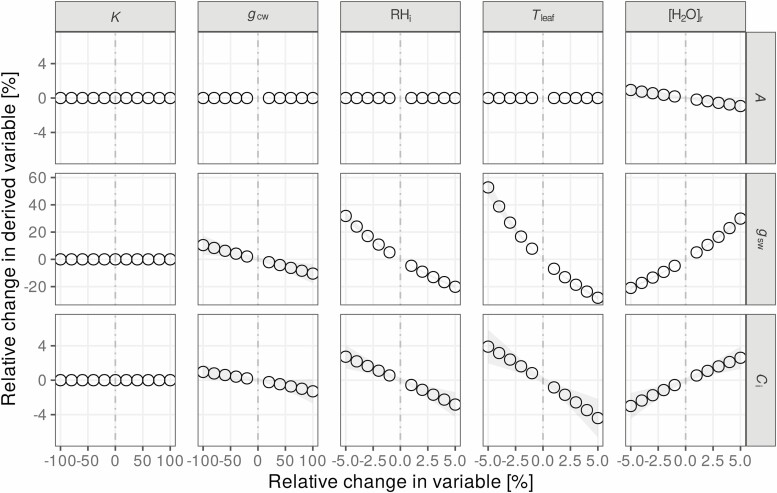
Effect of a relative change in measured or assumed variables on derived quantities. *A* is the net assimilation, *g*_sw_ is the stomatal conductance to water vapour, *C*_i_ is the mole fraction of CO_2_ in the substomatal air. *K* is the top-to-bottom ratio of stomatal conductance (default value: 0.5), *g*_cw_ is the cuticular conductance to water (default: 25 mmol m^−2^ s^−1^), the cuticular conductance to CO_2_ (*g*_cc_) was assumed to scale with this value as *g*_cc_ = *g*_cw_/20, as in Márquez *et al.* (2021) and Lamour *et al.* (2022), RH_i_ is the relative humidity in the stomatal cavities (default: 95 %, see Wong *et al.* 2022). *T*_leaf_ is the leaf temperature (defaults to the measured value of 25 °C), and [H_2_O]_r_ is the reference mole fraction for water measured by the gas analyser (default: 21 mmol mol^−1^). The vertical dash-dotted lines indicate the default values (0 % change). Averages of three different leaves are shown with standard deviations indicated by the grey-shaded area.

The results of the sensitivity analysis are presented in Fig. 4. Note that the parameter *K* does not reflect the relative gas flux contribution from each leaf surface. In gas-exchange chambers, the flux from the top and bottom halves is combined under the assumption that such mixing does not significantly influence the determination of *A*, *g*_sw_ and *C*_i_ (but see Márquez *et al.* 2023). Thus, *K* is only used in the calculations to weigh the potential effect of the upper and lower side boundary layer conductance on the total conductance to water vapour (LI-COR Biosciences Inc. 2022*a*). The boundary layer conductance in commercially available chambers is usually very large relative to typical stomatal conductances (21 times larger in this case), and this explains why this variable had only a negligible effect on the derived quantities. Similarly, the impact of cuticular conductance on gas-exchange fluxes is proportional to the ratio of the cuticular conductance to the total leaf conductance (Márquez *et al.* 2021). The highest *g*_cw_ used for the analysis in Fig. 4 (50 mmol m^−2^ s^−1^) was still somewhat less than 20 % of the total leaf conductance, confirming a previous analysis that showed that the effect of cuticular conductance only becomes significant when stomatal conductance is low (Lamour *et al.* 2022). The effect of unsaturation in the substomatal cavities may be more important: at the lowest value (RH_i_ = 90 %), *g*_sw_ was increased by over 30 % relative to the default (RH_i_ = 95 %), although the effect on *C*_i_ remained below 5 %. The largest observed effect was related to a bias in the measurement of leaf temperature. A 5 % scale calibration error in H_2_O mole fractions surprisingly affected *C*_i_ and *g*_sw_ less than a 5 % error in the measurement of leaf temperature, highlighting the importance of measuring leaf temperature correctly (Garen *et al.* 2022).

With the R package described here, similar tests can be integrated into analysis workflows, and used to show that the reported results are robust in the presence of a reasonable uncertainty. In addition, such an analysis can be used to show whether or not accounting for additional details (such as considering cuticular conductance) in the calculation of derived parameters is likely to have a significant effect on the variables of interest.

### Isotope data and mesophyll conductance

Gas-exchange data are often complemented by additional measurements, like, for example, an analysis of the isotopic composition of the gas fluxes. Such combined datasets can be used to derive mesophyll conductance (*g*_m_) by using a mathematical description of photosynthetic isotope discrimination (Evans *et al.* 1986; Pons *et al.* 2009; Tazoe *et al.* 2009, 2011; Farquhar and Cernusak 2012). From individual measurements of gas-exchange variables and isotope composition (the so-called ‘point method’), *g*_m_ can be calculated by assuming that the observed discrimination against ^13^CO_2_ equals the model-predicted discrimination (*g*_m_ is an unknown variable in this model). Alternatively, if measurements over a range of light or CO_2_ concentrations are available, a linear or non-linear regression approach can be used to estimate *g*_m_ (Evans *et al.* 1986; Tazoe *et al.* 2009). This ‘slope method’ not only makes the assumption that *g*_m_ stays constant over this range but also allows for the estimation of additional unknowns in the isotope model, such as the effective isotope fractionation related to respiration, *e*ʹ (Tazoe *et al.* 2009).

An assumption underlying the original model describing the relationship between carbon isotope discrimination in relation to CO_2_ concentrations was that the carbon pool of respiration and assimilation was shared (‘connected’). There is evidence that this is not completely correct (Pärnik and Keerberg 2007; Tcherkez *et al.* 2011; Nogués *et al.* 2014), and recently the isotope model was revised (Busch *et al.* 2020) based on the assumption that both carbon pools are disconnected. Busch *et al.* (2020) showed that using the disconnected model led to more robust *g*_m_ estimates under a range of light intensities and CO_2_ concentrations. gasanalyzer includes both models to allow researchers to test the effect of these assumptions on the derived *g*_m_.

Since commercially available gas analysers do not provide information on the isotopic composition of the used gasses, such data need to be acquired using different instruments and manually combined with the gas-exchange data before *g*_m_ values can be calculated using gasanalyzer. Physiological constants, such as the respiration in the light (*R*_L_) and the CO_2_ compensation point in the absence of mitochondrial respiration (Γ^*^), need to be provided. In addition, calculations using the isotopic model contain several fractionation factors describing how the abundance ratio of the carbon isotopes will change depending on physiological or physical processes. Some uncertainty exists regarding the exact value of several of these factors. For example, it has been shown that the value assumed for the fractionation factor associated with photorespiration (*f*) may strongly influence the calculation of the mesophyll conductance (Tazoe *et al.* 2009; Evans and von Caemmerer 2013). Estimates for *f* range between 7 ‰ and 16 ‰, and there is some disagreement about which value should be used (Ghashghaie *et al.* 2003; Igamberdiev *et al.* 2004; Tcherkez 2006; Lanigan *et al.* 2008; Evans and von Caemmerer 2013; Busch *et al.* 2020). One possible reason for the variability in the estimates is that the *in vivo* estimation of *f* typically assumes no effect of photorespiration on the mesophyll conductance, which is not necessarily true (Tholen *et al.* 2012, 2014). Additional uncertainty exists for the effective fractionation effect related to respiration (*e*ʹ; Ghashghaie *et al.* 2003; Busch *et al.* 2020).

**Figure 5.**
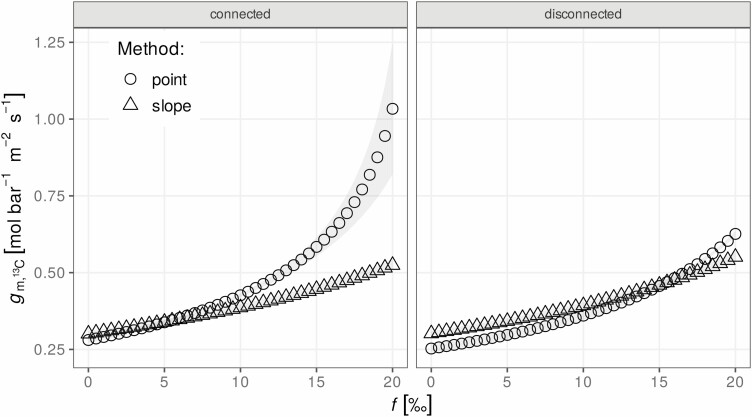
Comparison of the effect of the photorespiratory fractionation (*f*) on the estimates of mesophyll conductance (*g*_m,13C_) under different model assumptions. ‘connected’ and ‘disconnected’ refer to whether or not respiratory carbon pools are shared (sensu Busch *et al.* 2020). ‘slope’ and ‘point’ refer to different experimental approaches for estimating *g*_m_ (as described by Pons *et al.* 2009).

The considerations given above highlight the importance of testing model assumptions (such as connected vs. disconnected respiratory carbon pools) and assumed key variables (such as the values of *e*ʹ and *f*) when deriving *g*_m_ using combined measurements of gas exchange and isotope ratios. In the following example, previously published data (Tholen *et al.* 2012) from a tobacco leaf were used. By adjusting the CO_2_ in the leaf chamber (*C*_a_), *C*_i_ was kept constant during the measurements, and light was varied to obtain a range of *A*/*C*_a_ values. This allows calculation of *g*_m_ using both the slope and point methods. To account for the effect that respiration predominately releases carbon that was fixed before the start of the gas-exchange experiment, an effective *e*ʹ (Wingate *et al.* 2007) was calculated from the difference between the isotope ratio of the growth air and that of the tank air used for the experiments (Tholen *et al.* 2012; Evans and von Caemmerer 2013). An alternative correction was proposed more recently (Busch *et al.* 2020), and is implemented in gasanalyzer, but could not be used for this analysis since discrimination against ^13^C under growth conditions was not quantified for these plants. Fortunately, for the slope method, a non-linear fitting approach (Tazoe *et al.* 2009) can be used to make the *g*_m_ estimates free of assumptions regarding the exact value of *e*ʹ. To illustrate the usefulness of gasanalyzer in analysing gas-exchange data combined with measurements of the isotopic composition of the gas, a sensitivity analysis of the effect of *f* on *g*_m_ estimates was performed (Fig. 5). The difference between the ‘slope’ and ‘point’ methods and between ‘connected’ and ‘disconnected’ model assumptions were analysed.

Interestingly, the slope method provided more robust estimates of *g*_m_, which were not affected much by the model assumptions (connected or disconnected carbon pools) and although higher values of *f* increased the estimated *g*_m_, the effect was much smaller when the often-used point method is used with the disconnected model.

## Conclusion

The R package gasanalyzer provides a tool to import, pre-process and analyse data from gas-exchange instruments. It offers a reference implementation to calculate physiologically relevant variables from raw gas-exchange data and provides a framework with which to perform sensitivity analyses on such data. The package fills an important gap in reproducible data workflows by making it possible to automate all calculations from raw data to final results.

The provided examples demonstrated how to use the software package and illustrated the effects that biases or uncertainties in measurements, assumed values, or model assumptions can have on the conclusions drawn from gas-exchange measurements. Technical advances related to more accurate temperature measurements are likely to have far greater potential to improve gas-exchange instruments compared with accounting for differences in stomatal conductances between both sides of the leaves. Nevertheless, the examples shown here are based on a few datasets, and the results are not applicable to all possible conditions. It is recommended that researchers include similar analyses when evaluating data related to photosynthetic measurements, to gain more insight into the robustness of reported results and conclusions.

## Supporting Information

The following additional information is available in the online version of this article –

Supplementary Table 1. An overview of all variables used by the gasanalyzer package, including the default units, the commonly used mathematical symbols, corresponding names used by several gas-exchange instruments and a short description of the variables. Note that if no unit is applicable, the units column lists the R data type.

plae035_suppl_Supplementary_Materials

## Data Availability

All data and computer code used in this article are available at https://gitlab.com/plantphys/gasanalyzer.
