# Supplementary material for: GasanalyzeR: advancing reproducible research using a new R package for photosynthesis data workflows
Source: AoB Plants. 2024 Jun 20;16(4):plae035. doi: 10.1093/aobpla/plae035 (PMC11261163; doi:10.1093/aobpla/plae035)
Supplement: plae035_suppl_Supplementary_Materials [file plae035_suppl_supplementary_materials.pdf]

Supporting Information

This document contains additional information on the variables used by the `gasanalyzer` R package. The latest version of this file may be found at the GitLab repository.

Table S1: An overview of all variables used by the `gasanalyzer` package, including the default units, commonly-used mathematical symbols, corresponding names used by several gas-exchange instruments, and short descriptions of the variables. Note that if no unit is applicable, the units column lists the `R` data type.

name	units	symbol	CIRAS4	GFS3000	Li6400	Li6800	description
SysObs.Obs	integer				Obs	Obs	Observation number
SysObs.Time	posix		TIMESTAMP			time	Unix time stamp (seconds since 00:00:00 UTC on 1 January 1970) of the current observation
SysObs.Elapsed	s				FTime	elapsed	Seconds since first observation
SysObs.Date	character			Date		date	Date in local timezone of the current observation as character string
SysObs.HHMMSS	character			Time	HHMMSS	hhmmss	Time in local timezone of the current observation as character string
SysObs.Averaging	s					averaging	Additional averaging on current observation
SysObs.Instrument	character						The instrument type used to collect this data
SysObs.Serial	character						The serial number of the instrument
SysConst.AvgTime	s					AvgTime	Averaging time for measurements
SysConst.Geometry	character					Geometry	Text string specifying chamber geometry, used for calculating GasEx.gbw
SysConst.UserCal	list						List containing user calibration
SysConst.FactCal	list						List containing factory calibration
SysConst.UseDynamic	logical					UseDynamic	Specifies whether or not to use non-steady state equations
SysConst.TleafOffset	°C					Offset	Offset correction for Tleaf
SysConst.Tleaf2Offset	°C					Offset2	Offset correction for Tleaf2
Const.S	cm^2	S	Aleaf	Area	Area	S	Leaf area
Const.K	numeric	K	StomataR		StmRat	K	Stomatal ratio
Const.CustomBLC	mol m^{-2} s^{-1}				BLC_1	Custom	One-sided boundary layer conductance
Const.gcw	mol m^{-2} s^{-1}	g_{cw}					User-specified cuticular conductance to water vapor
Const.gcc	mol m^{-2} s^{-1}	g_{cc}					User-specified cuticular conductance to CO2
Const.RL	µmol m^{-2} s^{-1}	R_L					User-specified mitochondrial respiration in the light
Const.GammaStar	µmol mol^{-1}	Γ^*					User-specified CO2 photocompensation point (as mole fraction!)
Const.RHi	%	RH_i					User-specified relative humidity in the substomatal cavity.
Const.Oxygen	%	$[O_2]$		Oxygen	Oxygen%	Oxygen	Oxygen mole fraction in the chamber
Const.fPS2	numeric	β			PS2/1	PS2/1	Fraction of energy partitioned to PS2
GasEx.Time	posix					TIME	Unix time stamp for the measurement
GasEx.E	mol m^{-2} s^{-1}	E		E	Trans	E	Transpiration rate
GasEx.Emm	mmol m^{-2} s^{-1}	E	E		Trmmol	Emm	Transpiration rate in mmol per square meter per second
GasEx.A	µmol m^{-2} s^{-1}	A	A	A	Photo	A	Net assimilation rate
GasEx.Ca	µmol mol^{-1}	C_a				Ca	CO2 mole fraction in ambient air
GasEx.Cs	µmol mol^{-1}	C_s					CO2 mole fraction at the leaf surface
GasEx.Ci	µmol mol^{-1}	C_i	Ci	ci	Ci	Ci	CO2 mole fraction in intercellular airspaces
GasEx.pCi	Pa	p_{C_i}			Ci_Pa	Pci	Intercellular CO2 in partial pressure units
GasEx.pCa	Pa	p_{C_a}				Pca	Ambient CO2 in partial pressure units
GasEx.gsw	mol m^{-2} s^{-1}	g_{sw}	gs		Cond	gsw	Stomatal conductance to water vapor
GasEx.gbw	mol m^{-2} s^{-1}	g_{bw}				gbw	Boundary layer conductance to water vapor
GasEx.gtc	mol m^{-2} s^{-1}	g_{tc}			CndCO2	gtc	Total conductance to CO2

Table S1: An overview of all variables used by the `gasanalyzer` package, including the default units, commonly-used mathematical symbols, corresponding names used by several gas-exchange instruments, and short descriptions of the variables. Note that if no unit is applicable, the units column lists the `R` data type. *(continued)*

name	units	symbol	CIRAS4	GFS3000	Li6400	Li6800	description
GasEx.gtw	mol m^{-2} s^{-1}	g_{tw}		GH2O	CndTotal	gtw	Total conductance to water vapor
GasEx.Rabs	W m^{-2}	R_{abs}			R(W/m2)	Rabs	Absorbed short wave radiation contribution to the energy balance
GasEx.TleafEB	°C	$T_{leaf,EB}$				TleafEB	Leaf temperature from energy balance
GasEx.TleafCnd	°C	$T_{leaf,Cnd}$			CTleaf	TleafCnd	Leaf temperature used for gas-exchange computations
GasEx.TairCnd	°C	$T_{air,Cnd}$			CTair		Air temperature used for gas-exchange computations
GasEx.SVPleaf	kPa	SVP_{leaf}			SVTleaf	SVPleaf	Saturation vapor pressure at leaf temperature
GasEx.RHcham	%	RH_{cham}	RH	rh	RH_S	RHcham	Relative humidity in the chamber
GasEx.VPcham	kPa	VP_{cham}			vapS_kPa	VPcham	Vapor pressure in the chamber
GasEx.SVPcham	kPa	SVP_{cham}			SVTair	SVPcham	Saturation vapor pressure in the chamber
GasEx.VPDleaf	kPa	VPD_{leaf}	VPD		VpdL	VPDleaf	Vapor pressure deficit at leaf temperature
GasEx.LatHFlux	W m^{-2}	E_l				LatHFlux	Latent heat flux contribution to the energy balance
GasEx.SenHFlux	W m^{-2}	H_l				SenHFlux	Sensible Heat Flux contribution to the energy balance
GasEx.NetTherm	W m^{-2}					NetTherm	Net thermal contribution to the energy balance
GasEx.EBSum	W m^{-2}					EBSum	Sum of all energy balance components
GasEx.Asty	µmol m^{-2} s^{-1}	A_{sty}				Asty	Net assimilation rate calculated using steady state equations
GasEx.Esty	mmol m^{-2} s^{-1}	E_{sty}				Esty	Transpiration rate calculated using steady state equations
Dynamic.Adyn	µmol m^{-2} s^{-1}	A_{dyn}				Adyn	Net assimilation rate, calculated for non-steady state conditions
Dynamic.Crd	µmol mol^{-1}	C_{rd}		CO2buf		Crd	Non-steady state, dry-equivalent reference CO2
Dynamic.Csd	µmol mol^{-1}	C_{sd}				Csd	Non-steady state, dry-equivalent sample CO2
Dynamic.dCsd_dt	µmol mol^{-1} s^{-1}	dC_{sd}/dt				dCsd/dt	Rate of change of Csd
Dynamic.alphaVc	cm^3	αV_c				αVc	Effective CO2 Volume of the gas-exchange chamber
Dynamic.Edyn	mmol m^{-2} s^{-1}	E_{dyn}				Edyn	Evapotranspiration calculated for non-steady state conditions
Dynamic.Hr	mmol mol^{-1}	H_r				Hr	Non-steady state reference H2O
Dynamic.Hs	mmol mol^{-1}	H_s				Hs	Non-steady state sample H2O
Dynamic.dHs_dt	mmol mol^{-1} s^{-1}	dH_s/dt				dHs/dt	Rate of change of Hs
Dynamic.alphaVh	cm^3	αV_h				αVh	Effective H2O Volume
LeafQ.Qabs	µmol m^{-2} s^{-1}	Q_{abs}				Qabs	PPFD absorbed by the leaf
LeafQ.Qin	µmol m^{-2} s^{-1}	Q_{in}				Qin	PPFD incident on the leaf
LeafQ.alpha	numeric	α			LeafAbs	alpha	PPFD absorptance
LeafQ.Conv	J µmol^{-1}					convert	Conversion from PPFD to Watts per square meter
Leak.Leak	µmol s^{-1}					Leak	Leakage from chamber
Leak.LeakPct	%					LeakPct	Leakage as percent of flow
Leak.CorrFact	numeric					CorrFact	Leak correction factor
Leak.CorrFactPct	%					CorrFactPct	Leak correction factor as percent
Leak.Fan	µmol s^{-1}					Fan	Estimated mixing fan flow
FLR.Fo	numeric	F_o	Fo	Fo	Fo	Fo	Dark-adapted, minimal fluorescence
FLR.Fm	numeric	F_m	Fm	Fm	Fm	Fm	Dark-adapted maximal fluorescence
FLR.Fv_Fm	numeric	F_v/F_m	FvFm	Fv/Fm	Fv/Fm	Fv/Fm	Maximum quantum efficiency of PSII photochemistry
FLR.Adark	µmol m^{-2} s^{-1}				Adark	A_dark	Dark assimilation rate before Fm
FLR.Fs	numeric	F_s	Fs	F	Fs	Fs	Steady state fluorescence
FLR.Fmp	numeric	F'_m	FmP	Fm'	Fm'	Fm'	Light-adapted maximal fluorescence
FLR.phiPS2	numeric	Φ_{PSII}	phiPSII	Yield	PhiPS2	PhiPS2	Operating quantum efficiency of Photosystem II in the light
FLR.QabsFs	µmol m^{-2} s^{-1}	$Q_{abs,Fs}$				Qabs_fs	Light quanta absorbed at Fs
FLR.AFs	µmol m^{-2} s^{-1}	A_{Fs}				A_fs	Net assimilation rate at Fs
FLR.ETR	µmol m^{-2} s^{-1}	J_F	J	ETR	ETR	ETR	Electron transport rate calculated from chlorophyll fluorescence
FLR.phiCO2	mol mol^{-1}	Φ_{CO2}			PhiCO2	PhiCO2	Quantum efficency of the carboxylation rate, based on absorbed irradiance
FLR.NPQ	numeric		NPQ	NPQ	NPQ	NPQ	Non-photochemical quenching
FLR.FopAlt	numeric	$F'_{o,alt}$		Fo'calc		alt. Fo'	Alternative estimate for Fo' : Fo/(Fv/Fm+Fo/Fm')

Table S1: An overview of all variables used by the `gasanalyzer` package, including the default units, commonly-used mathematical symbols, corresponding names used by several gas-exchange instruments, and short descriptions of the variables. Note that if no unit is applicable, the units column lists the `R` data type. *(continued)*

name	units	symbol	CIRAS4	GFS3000	Li6400	Li6800	description
FLR.Fop	numeric	F'_o	FoP	Fo'	Fo'	Fo'	Light-adapted minimal fluorescence
FLR.Fvp_Fmp	numeric	F'_v/F'_m	FvFmP		Fv'/Fm'	Fv'/Fm'	Maximum quantum efficiency of PSII in the light, if all reaction-centres were open
FLR.qP	numeric	q_P	qP	qP	qP	qP	Photochemical quenching coefficient
FLR.qPFo	numeric	$q_{P,Fo}$				qP_Fo	Photochemical quenching coefficient, using Fo instead of Fo'
FLR.qN	numeric	q_N	qNP		qN	qN	Non-photochemical quenching coefficient
FLR.qNFo	numeric	$q_{N,Fo}$				qN_Fo	Non-photochemical quenching coefficient, using Fo instead of Fo'
FLR.qL	numeric	q_L	qL	qL		qL	An estimate for the fraction of open PSII centres
FLR.QinFs	numeric	$Q_{in,Fs}$			ParInFs		Light intensity measured at Fs
FLR.PhiQin_4	µmol m^{-2} s^{-1}	$\Phi_{PSII} Q_{in}/4$					A convenience parameter for some analyses. PhiPS2 x Qin/4
FLR.gm	mol m^{-2} Pa^{-1} s^{-1}	$g_{m,flr}$					Mesophyll conductance derived from fluorescence
FLR.Cc	µmol mol^{-1}	$C_{c,flr}$					Chloroplast CO2 mole fraction derived from fluorescence
FlrStats.Favg	numeric	F_{avg}			Fmean	F_avg	Average fluorescence
FlrStats.dF_dt	min^{-1}	dF/dt			dF/dt	dF/dt	Rate of change of the fluorescence
FlrStats.Period	s					period	Period used for calculating statistics
d13CConst.ab	‰	a_b					13C fractionation associated with diffusion through air boundary layers
d13CConst.as	‰	a_s					13C fractionation associated with free diffusion through air
d13CConst.am	‰	a_m					13C fractionation associated with diffusion through liquid
d13CConst.b	‰	b					13C fractionation associated with carboxylation
d13CConst.e	‰	e					13C fractionation associated with mitochondrial respiration
d13CConst.f	‰	f					13C fractionation associated with photorespiration
d13CConst.delta13CO2g	‰	$\delta^{13}C{-}CO_{2,g}$					Isotopic composition of CO2 in the air during growth conditions
d13CConst.Deltag	‰	Δ_o^{growth}					Observed discrimination against 13CO2, measured under ambient growth conditions
d13CMeas.delta13CO2s	‰	$\delta^{13}C{-}CO_{2,s}$					Isotopic composition of sample CO2
d13CMeas.delta13CO2r	‰	$\delta^{13}C{-}CO_{2,r}$					Isotopic composition of the reference CO2
d13C.xi	numeric	ξ					The inverse of the relative drawdown of dry-equivalent CO2 in the leaf chamber
d13C.ap	‰	a'					Weighted 13C fractionation associated with air and boundary layers
d13C.ep	‰	e'					Corrected fractionation associated with respiration
d13C.t	numeric	t					A ternary correction factor
d13C.Deltai	‰	Δ_i					Model-predicted discrimination against 13CO2
d13C.Deltao	‰	Δ_o					Observed discrimination against 13CO2
d13C.DeltaiDeltao	‰	$\Delta_i - \Delta_o$					Difference between predicted and observed discrimination against 13CO2
d13C.A_pCa	µmol m^{-2} Pa^{-1} s^{-1}	A/p_{C_a}					Ratio between net assimilation and the partial pressure of CO2 in the ambient air
d13C.gm	mol m^{-2} Pa^{-1} s^{-1}	$g_{m,{}^{13}C}$					Mesophyll conductance derived from carbon isotope discrimination
d13C.Cc	µmol mol^{-1}	$C_{c,{}^{13}C}$					Chloroplast CO2 derived from carbon isotope discrimination
Meas.Time	posix	$Time$				TIME	Unix time stamp for the measurement
Meas.Flow	µmol s^{-1}	$Flow$		Flow	Flow	Flow	Flow rate into the chamber
Meas.Pa	kPa	P_a	Patm	Pamb	Press	Pa	Atmospheric pressure
Meas.DeltaPcham	kPa	ΔP_{cham}				ΔPcham	Chamber overpressure
Meas.Tamb	°C	T_{amb}	Tamb	Tamb			Ambient temperature sensor
Meas.Tair	°C	T_{air}	Tcuv	Tcuv	Tair	Tair	Chamber air temperature
Meas.Tleaf	°C	T_{leaf}	Tleaf	Tleaf	Tleaf	Tleaf	Leaf thermocouple 1
Meas.Tleaf2	°C	T_{leaf2}				Tleaf2	Leaf thermocouple 2
Meas.FanSpeed	numeric			Imp		Fan_speed	Chamber fan speed
Meas.QambIn	µmol m^{-2} s^{-1}	$Q_{amb,in}$	PARi	PARtop	PARi	Qamb_in	In-chamber ambient PPFD
Meas.QambInBot	µmol m^{-2} s^{-1}	$Q_{amb,in,bot}$		PARbot			In-chamber ambient PPFD from the bottom
Meas.QambOut	µmol m^{-2} s^{-1}	$Q_{amb,out}$	PARe	PARamb	PARo	Qamb_out	External ambient PPFD
Meas.CO2s	µmol mol^{-1}	$[CO_2]_s$	CO2a	ca	CO2S	CO2_s	CO2 mole fraction in wet air leaving the chamber
Meas.CO2a	µmol mol^{-1}	$[CO_2]_a$				CO2_a	CO2 mole fraction in wet air leaving the chamber, before match correction

Table S1: An overview of all variables used by the `gasanalyzer` package, including the default units, commonly-used mathematical symbols, corresponding names used by several gas-exchange instruments, and short descriptions of the variables. Note that if no unit is applicable, the units column lists the `R` data type. *(continued)*

name	units	symbol	CIRAS4	GFS3000	Li6400	Li6800	description
Meas.CO2r	µmol mol^{-1}	$[CO_2]_r$	CO2r	CO2abs	CO2R	CO2_r	CO2 mole fraction in wet air entering the chamber
Meas.H2Os	mmol mol^{-1}	$[H_2O]_s$	H2Oa	wa	H2OS	H2O_s	H2O mole fraction in air leaving the chamber
Meas.H2Oa	mmol mol^{-1}	$[H_2O]_a$				H2O_a	H2O mole fraction in air leaving the chamber, before match correction
Meas.H2Or	mmol mol^{-1}	$[H_2O]_r$	H2Or		H2OR	H2O_r	H2O mole fraction in air entering the chamber
Meas2.TdR	°C	$T_{d,r}$			TdR	Td_r	Reference cell dew point
Meas2.TdS	°C	$T_{d,s}$			TdS	Td_s	Sample cell dew point
MchEvent.Time	posix					time	Unix time stamp of the last match
MchEvent.HHMMSS	character					hhmmss	Time of last match as character string
MchEvent.CO2time	posix					co2_t	Unix time stamp of the last CO2 match
MchEvent.H2Otime	posix					h2o_t	Unix time stamp of the last H2O match
MchEvent.Count	integer					count	Match count number
MchEvent.CO2adj	µmol mol^{-1}					co2_adj	CO2r - CO2s at last point match
MchEvent.H2Oadj	mmol mol^{-1}					h2o_adj	H2Or - H2Os at last point match
MchEvent.CO2pMatch	µmol mol^{-1}				CsMch	co2_match	CO2r - CO2a at last point match
MchEvent.H2OpMatch	mmol mol^{-1}				HsMch	h2o_match	H2Or - H2Oa at last point match
MchEvent.CO2at	µmol mol^{-1}				matchCO2	co2_at	CO2 at last match
MchEvent.H2Oat	mmol mol^{-1}				matchH2O	h2o_at	H2O at last match
MchStatus.MatchCO2	µmol mol^{-1}					MatchCO2	Currently used CO2 match adjustment value
MchStatus.MatchH2O	mmol mol^{-1}					MatchH2O	Currently used H2O match adjustment value
MchStatus.CFaCO2	numeric					cf_co2_a	CO2 match coefficient
MchStatus.CFbCO2	numeric					cf_co2_b	CO2 match coefficient
MchStatus.CFcCO2	numeric					cf_co2_c	CO2 match coefficient
MchStatus.CFdCO2	numeric					cf_co2_d	CO2 match coefficient
MchStatus.CFaH2O	numeric					cf_h2o_a	H2O match coefficient
MchStatus.CFbH2O	numeric					cf_h2o_b	H2O match coefficient
MchStatus.CFcH2O	numeric					cf_h2o_c	H2O match coefficient
MchStatus.CFdH2O	numeric					cf_h2o_d	H2O match coefficient
MchStatus.CO2fitLow	µmol mol^{-1}					co2_fit_low	CO2 range match fit lower limit
MchStatus.CO2fitHigh	µmol mol^{-1}					co2_fit_high	CO2 range match fit upper limit
MchStatus.H2OfitLow	mmol mol^{-1}					h2o_fit_low	H2O range match fit lower limit
MchStatus.H2OfitHigh	mmol mol^{-1}					h2o_fit_high	H2O range match fit upper limit
MchStatus.Status	character						Free-text match status information
FlrLS.Q	µmol m^{-2} s^{-1}					Q	Fluorometer photon flux density
FlrLS.fred	1					f_red	Fraction red
FlrLS.fgreen	1						Fraction green
FlrLS.fblue	1					f_blue	Fraction blue
FlrLS.fwhite	1						Fraction white
FlrLS.ffarred	1					f_farred	Fraction far red
FlrLS.F	numeric		F		F	F	Demodulated fluorescence
FlrLS.Qmodavg	µmol m^{-2} s^{-1}					Q_modavg	Modulated contribution to FlrLS.Q
FlrLS.Qred	µmol m^{-2} s^{-1}					Q_red	Red actinic contribution to FlrLS.Q
FlrLS.Qblue	µmol m^{-2} s^{-1}					Q_blue	Blue actinic contribution to FlrLS.Q
FlrLS.Qfarred	µmol m^{-2} s^{-1}					Q_farred	Far red contribution to FlrLS.Q
FlrLS.State	numeric					state	Specifies flash type: 0=Normal, 1=Rect, 2=MPF, 3=Ind, 4=Dark
FlrLS.Status	character		Status.1				Additional free text status information related to the fluorometer
HeadLS.Q	µmol m^{-2} s^{-1}					Q	Head light source output
HeadLS.fred	1		Red			f_red	Head light source, fraction red
HeadLS.fgreen	1		Green			f_green	Head light source, fraction green

Table S1: An overview of all variables used by the `gasanalyzer` package, including the default units, commonly-used mathematical symbols, corresponding names used by several gas-exchange instruments, and short descriptions of the variables. Note that if no unit is applicable, the units column lists the `R` data type. *(continued)*

name	units	symbol	CIRAS4	GFS3000	Li6400	Li6800	description
HeadLS.fblue	1		Blue			f_blue	Head light source, fraction blue
HeadLS.fwhite	1		White			f_white	Head light source, fraction white
HeadLS.ffarred	1		FarRed				Head light source, fraction far red
ConsoleLS.Q	µmol m^{-2} s^{-1}					Q	Console light source intensity
ConsoleLS.fred	1					f_red	Console light source, fraction of red light
ConsoleLS.fgreen	1					f_green	Console light source,fraction of green light
ConsoleLS.fblue	1					f_blue	Console light source, fraction of blue light
ConsoleLS.fwhite	1					f_white	Console light source, fraction of white light
Stability.Stable	integer				State	Stable	Number of stable variables
Stability.Total	integer					Total	Number of variables tracked
Stability.State	character					State	stable variables / total variables
Raw.CO2aAbs	numeric					abs_c_a	CO2 absorptance sample cell
Raw.CO2rAbs	numeric					abs_c_b	CO2 absorptance reference cell
Raw.H2OaAbs	numeric					abs_h_a	H2O absorptance sample cell
Raw.H2OrAbs	numeric					abs_h_b	H2O absorptance reference cell
Raw.H2Oa	mmol mol^{-1}						back-calculated H2O in mole fraction, sample cell
Raw.H2Or	mmol mol^{-1}			H2Oabs			back-calculated H2O in mole fraction, reference cell
Raw.CO2aAbsP	kPa^{-1}						back-calculated CO2 in absorptance per kPa, sample cell
Raw.CO2rAbsP	kPa^{-1}						back-calculated CO2 in absorptance per kPa, reference cell
Status.FlowS	µmol s^{-1}					Flow_s	Sample cell flow
Status.FlowR	µmol s^{-1}					Flow_r	Reference cell flow
Status.Txchg	°C	T_{xchg}			TBlk	Txchg	Heat exchanger temperature
Status.Tirga	°C	T_{irga}				Tirga	IRGA block temperature
Status.Ts	°C					Ts	A cell inlet temperature
Status.Tr	°C					Tr	B cell inlet temperature
Status.Status	character		Status	Status	Status		Free text status information
LeakConst.FsMeas	character					Fs_meas	Character string with measured FlowS adjustement
LeakConst.FsTrue	character					Fs_true	Character string with true FlowS adjustment
LeakConst.CFaFan	numeric					fan_a	Fan flow calibration coefficient
LeakConst.CFbFan	numeric					fan_b	Fan flow calibration coefficient
LeakConst.CFcFan	numeric					fan_c	Fan flow calibration coefficient
LeakConst.CFdFan	numeric					fan_d	Fan flow calibration coefficient
LeakConst.LeakWt	numeric					leak_wt	Leak weighting factor
ChambConst.Aperture	cm^2					Aperture	Aperture used for the chamber
ChambConst.PoBLC	kPa					blc_Po	Pressure for calculation of GasEx.gbw
ChambConst.CFaBLC	numeric					blc_a	LI6800 specific coefficient for GasEx.gbw
ChambConst.CFbBLC	numeric					blc_b	LI6800 specific coefficient for GasEx.gbw
ChambConst.CFcBLC	numeric					blc_c	LI6800 specific coefficient for GasEx.gbw
ChambConst.CFdBLC	numeric					blc_d	LI6800 specific coefficient for GasEx.gbw
ChambConst.CFeBLC	numeric					blc_e	LI6800 specific coefficient for GasEx.gbw
ChambConst.SmaxBLC	cm^2					blc_maxS	LI6800 specific max. area for GasEx.gbw calculation
ChambConst.SminBLC	cm^2					blc_minS	LI6800 specific min. area for GasEx.gbw calculation
ChambConst.SslopeBLC	numeric				BLCslope	blc_S_slope	LI6400 specific coefficient for GasEx.gbw
ChambConst.SoffsetBLC	numeric				BLCoffst	blc_S_offset	LI6400 specific coefficient for GasEx.gbw
LTConst.DeltaTw	K	ΔT_w				deltaTw	Wall-air temp difference
LTConst.fT1	1					fT1	Fraction of TleafCnd measured by Tleaf
LTConst.fT2	1					fT2	Fraction of TleafCnd measured by Tleaf2
LTConst.fTEB	1				EBal?	fTeb	Fraction of TleafCnd measured by TleafEB
QConst.fQambIn	1					fQ_Amb_in	Fraction of PPFD coming from in-chamber ambient light

Table S1: An overview of all variables used by the `gasanalyzer` package, including the default units, commonly-used mathematical symbols, corresponding names used by several gas-exchange instruments, and short descriptions of the variables. Note that if no unit is applicable, the units column lists the `R` data type. *(continued)*

name	units	symbol	CIRAS4	GFS3000	Li6400	Li6800	description
QConst.fQambOut	1					fQ_Amb_out	Fraction of PPFD coming from external ambient light
QConst.fQconsoleLS	1					fQ_ConsoleLS	Fraction of PPFD coming from a console light source
QConst.fQflr	1					fQ_Flr	Fraction of PPFD coming from the fluorometer
QConst.fQheadLS	1					fQ_HeadLS	Fraction of PPFD coming from the head light source
QConst.fQin	1				f_parin		Fraction of Meas.QambIn used for energy-balance calculations
QConst.fQout	1				f_parout		Fraction of Meas.QambOut used for energy-balance calculations
LQConst.Ambient	character					Ambient	Ambient light identifier
LQConst.Leaf	character					Leaf	Leaf type identifier
LQConst.AbsAmbient	numeric	$\alpha_{ambient}$		ETR-Fac		abs_ambient	Leaf absorptance for ambient
LQConst.BlueAbsFlr	numeric				BlueAbs	abs_blueFlr	Leaf absorptance for blueFlr
LQConst.BlueAbsLED	numeric					abs_blueLED	Leaf absorptance for blueLED
LQConst.GreenAbsLED	numeric					abs_greenLED	Leaf absorptance for greenLED
LQConst.RedAbsFlr	numeric				RedAbs	abs_redFlr	Leaf absorptance for redFlr
LQConst.RedAbsLED	numeric					abs_redLED	Leaf absorptance for redLED
LQConst.WhiteAbsLED	numeric					abs_whiteLED	Leaf absorptance for whiteLED
LQConst.ConvAmbient	J µmol^{-1}				alphaK	k_ambient	Converts Qin to Rabs for ambient
LQConst.BlueConvFlr	J µmol^{-1}					k_blueFlr	Converts Qin to Rabs for blueFlr
LQConst.BlueConvLED	J µmol^{-1}					k_blueLED	Converts Qin to Rabs for blueLED
LQConst.GreenConvLED	J µmol^{-1}					k_greenLED	Converts Qin to Rabs for greenLED
LQConst.RedConvFlr	J µmol^{-1}					k_redFlr	Converts Qin to Rabs for redFlr
LQConst.RedConvLED	J µmol^{-1}					k_redLED	Converts Qin to Rabs for redLED
LQConst.WhiteConvLED	J µmol^{-1}					k_whiteLED	Converts Qin to Rabs for whiteLED
Auxilarry.IO1	V			Aux1		I/O1	Auxillary analog IO
Auxilarry.IO2	V			Aux2		I/O2	Auxillary analog IO
Auxilarry.IO3	V					I/O3	Auxillary analog IO
Auxilarry.IO4	V					I/O4	Auxillary analog IO
Auxilarry.IO5	V					I/O5	Auxillary analog IO
Auxilarry.IO6	V					I/O6	Auxillary analog IO
Auxilarry.IO7	V					I/O7	Auxillary analog IO
Auxilarry.IO8	V					I/O8	Auxillary analog IO
UserDefVar.Object	integer			Object			User-defined object number
UserDefVar.Comment	character		Comment	Comment			User-defined free-text comment
gasanalyzer.UseEqUnits	logical						Specifies whether or not the latest calculations enforced units
gasanalyzer.Equations	list						A list of gas-exchange equations
gasanalyzer.Version	character						Version number of gasanalyzer used to generate this list
